# Epidemiology of clinically relevant *Entamoeba spp*. *(E*. *histolytica*/*dispar*/*moshkovskii/bangladeshi)*: A cross sectional study from North India

**DOI:** 10.1371/journal.pntd.0009762

**Published:** 2021-09-07

**Authors:** Aradhana Singh, Tuhina Banerjee, Uzma Khan, Sunit Kumar Shukla

**Affiliations:** 1 Department of Microbiology, Institute of Medical Sciences, Banaras Hindu University, Varanasi, India; 2 Department of Applied microbiology, Institute of Science, Banaras Hindu University, Varanasi, India; 3 Department of Gastroenterology, Institute of Medical Sciences, Banaras Hindu University, Varanasi, India; NIH-National Institute for Research in Tuberculosis-ICER, INDIA

## Abstract

**Background:**

*Entamoeba* infections have major impact on millions of the people worldwide. *Entamoeba histolytica* has long been accepted as the only pathogenic species. However, recent reports of other *Entamoeba* spp. in symptomatic cases have raised questions on their pathogenicity.

**Methodology/Principal findings:**

Total 474 stool samples and 125 liver aspirates from patients with intestinal and extra intestinal manifestations and from community were included. Sewage samples from the hospital and the city were also included. Microscopic examination and molecular detection were performed to detect presence of *E*. *histolytica/ dispar*/ *moshkovskii/ bangladeshi*. The associated demographic and socioeconomic factors were statistically analyzed with the presence of *Entamoeba*. Microscopy detected *Entamoeba* spp. in 5.4% stool and 6.4% liver aspirate samples. Through nested multiplex PCR, prevalence of *Entamoeba* spp. in intestinal and extra-intestinal cases was 6.6% (20/301) and 86.4% (108/125) respectively and in asymptomatic population was 10.5% (13/123). Sewage samples did not show presence of any *Entamoeba* spp. Uneducated subjects, low economic conditions, untreated drinking water, consumption of raw vegetables and habit of not washing hands before meals were significantly associated with presence of *Entamoeba* spp.

**Conclusions:**

*E*. *histolytica* still remains the only *Entamoeba* spp. in invasive extra intestinal infections. *E*. *dispar* was detected in both asymptomatic and symptomatic intestinal infections. Routine identification of *Entamoeba* spp. should incorporate PCR based detection methods.

## Author summary

Amoebiasis, a neglected tropical disease, caused by *Entamoeba* sp., is one of the leading causes of death due to any parasitic disease worldwide and have a major impact on millions of people. There has been constant debate on the commensal and pathogen status of several *Entamoeba* spp., owing to sporadic reports of infections due to different *Entamoeba* species. In view of this, we conducted this study to provide detailed data of prevalence of the *Entamoeba* species through molecular method in different types of samples among the susceptible population in an endemic region. Despite the existence of specific and sensitive molecular techniques, microscopy because of its affordability and accessibility remains the diagnostic tool for the *Entamoeba* infections. Microscopy cannot differentiate other related species from *Entamoeba histolytica*. Thus, World Health Organization has recommended the use of advanced techniques in the diagnosis of *Entamoeba* infection. Further, the most common mode of *Entamoeba* infections is the ingestion of contaminated food and water. In this regard face to face interviews were carried out to know about the demographic details and household habits which can be a major force driving these infections. The establishment of such data clears the epidemiological status and conveys the proper control measures for better health care.

## Introduction

Infections due to *Entamoeba* spp. are ubiquitous. Alarmingly high prevalence is seen in tropical countries with lower socioeconomic communities and poor sanitation. [1] There has been constant debate on the commensal and pathogen status of several *Entamoeba* spp., owing to sporadic reports of infections due to *Entamoeba dispar* (*E*. *dispar)* and *Entamoeba moshkovskii* (*E*. *moshkovskii)*, which were once considered as commensals. [2,3] There are different species of *Entamoeba* found in humans of which *Entamoeba histolytica* (*E*. *histolytica)* is the one considered as pathogenic since the beginning. [4] It has been established that *E*. *histolytica* has the potential to cause both invasive and non-invasive infections while *E*. *dispar* and *E*. *moshkovskii* are associated with non-invasive infections only. [5] Recently, *Entamoeba bangladeshi* (*E*. *bangladeshi)* causing diarrhea in children has also been reported. [6] The epidemiological data from most of the resource limited endemic countries are based on microscopy with very low sensitivity. Consequently, differentiation between the morphologically identical *E*. *histolytica*, *E*. *dispar* and *E*. *moshkovskii* is often not possible. [7] A better way of reporting of the microscopic findings would be ‘stool samples positive for *E*. *histolytica/ dispar/ moshkovskii/ bangladeshi’* following the initial suggestions from the World Health Organization (WHO) for *E*. *histolytica/dispar*. [8]

The estimated prevalence of *Entamoeba* in humans by molecular methods of detection was found to be 3.55% worldwide. [9] The global mortality due to *E*. *histolytica* infections was reported to be 50–75 thousand deaths per year. [10] The clinical manifestations vary from asymptomatic colonization to amebic dysentery and invasive extra intestinal infections. Liver abscess is the most common extra intestinal presentation. [11] However, most of these global estimations are quite outdated and there is dearth of recent epidemiological data. Additionally, species like *E*. *dispar*, *E*. *moshkovskii* and *E*. *bangladeshi* have been a recent addition and therefore not much data exist on their prevalence. [2] It is widely acknowledged that *Entamoeba* infections are of public health importance but the geographical distribution and regional burden are yet to be determined. In this context, one of our recent study has shown high prevalence of *E*. *histolytica* in the cases of liver abscesses. [12] Thus, this study was conducted to provide an update on the current status of *Entamoeba* species distribution in different types of samples among the susceptible population in an endemic region in North India. Additionally, the factors associated with the presence of *Entamoeba* spp. were evaluated.

## Materials and methods

### Ethics statement

The study was ethically approved by Institute ethical committee, Institute of Medical Sciences, Banaras Hindu University, EC Registration No. ECR/526/Inst/UP/ 2014 Dt. 31.1.14 (Dean/2016-17/EC/045). Informed written consent was taken from all the patients prior to the study. In case of children, the informed written consent was taken from their parents/guardian.

### Study site and groups

**Fig 1:**
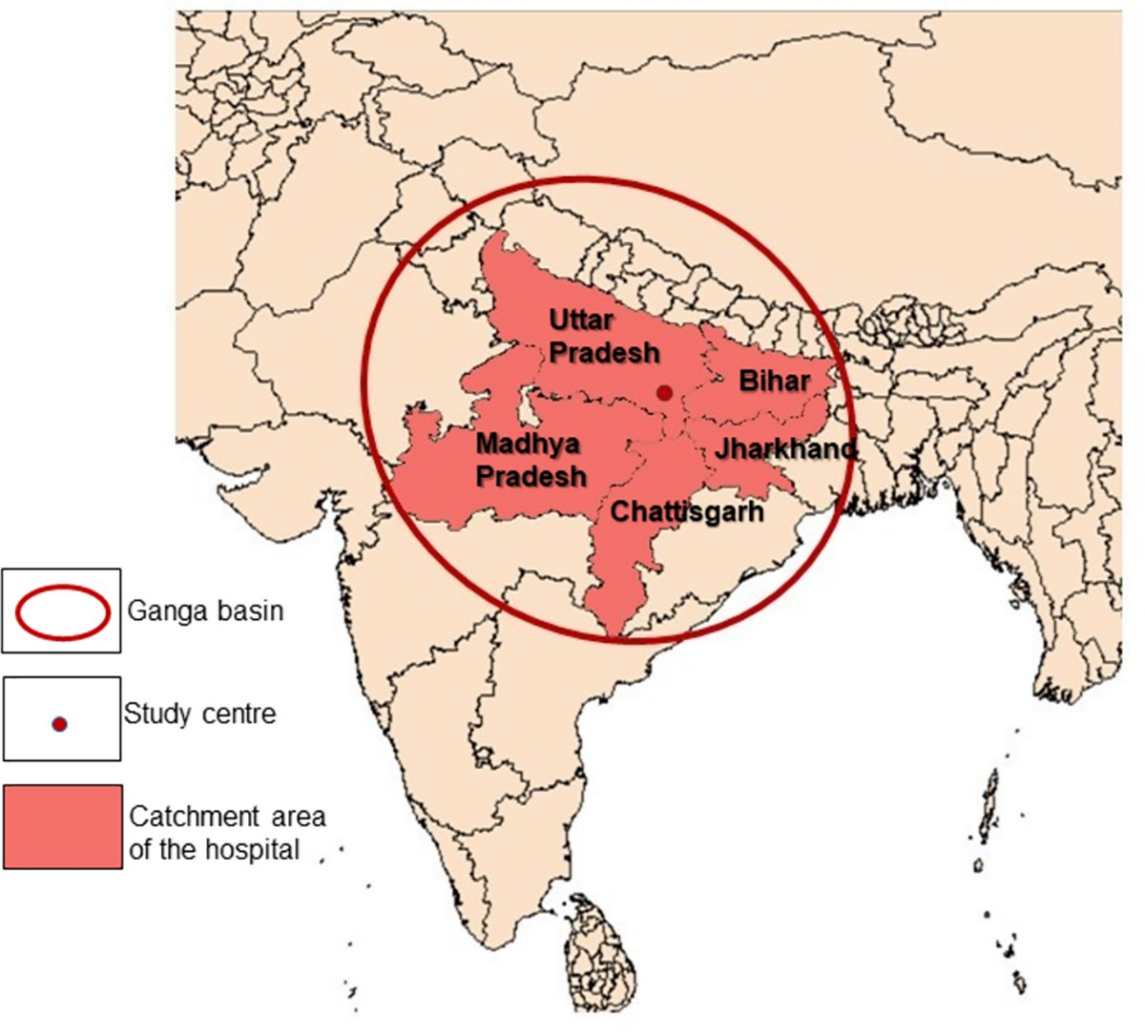
Catchment area of the hospital indicating the study centre and the Gangetic belt. Made with Natural Earth (link: https://www.naturalearthdata.com/downloads/10m-cultural-vectors/).

The present study was a hospital-based, cross-sectional study carried out from September 2016 to August 2019. The study included patients attending different out-patient departments of Sir Sunderlal hospital, Varanasi. The tertiary care hospital is a 2300 bedded referral hospital attending to the medical needs of approximately 150 million population from the states of Uttar Pradesh, Madhya Pradesh, Chhattisgarh, Bihar, Jharkhand and neighboring countries Bangladesh and Nepal. The catchment area of the hospital has been shown in Fig 1. These areas are endemic for parasitic infections like soil transmitted helminths and protozoal infections. [13] Factors such as tropical hot and humid weather conditions, high population density, street food culture, open air defecation and use of human and animal excreta as fertilizers in these regions further favor the transmission of the infections. Additionally, apparently healthy volunteers from an adjoining area (Naria, Varanasi) in the immediate vicinity (within 1 km) of the hospital were also included in the study. Written permission from the officer of the block was obtained. Thereafter, volunteers who agreed to participate in the study were included. The study population and choice of samples were divided based on intestinal and extra intestinal manifestations as well as asymptomatic cases. A pre-tested structured questionnaire was developed in English and Hindi about demographic details (i.e. age, gender), locality, education level, occupation, economic status (assessed by monthly income of the household) [14], behavioral risk like habit of handwashing before meals, living conditions including drinking water facility, consumption of raw vegetables and contact with domestic animals. Experienced research scholar collected the data in face-to-face interviews. For children, the questionnaire was filled by interviewing their parents/guardian.

### Inclusion criteria

The inclusion criteria for sample group 1 included stool samples from patients with complaints of gastrointestinal (GI) discomfort including diarrhea or dysentery with or without abdominal pain, mucous/blood in stool, abdominal distension, flatulence, nausea, vomiting, anal itch or bloating. For sample group 2, stool samples from human immunodeficiency virus (HIV) positive patients attending the anti-retro viral therapy (ART) centre of the associated tertiary care hospital with any or all of the above-mentioned GI discomfort complaints were included. Diarrhea was defined as at least three watery or loose stools in 24-hour period that lasted for a minimum of 3 days before sample collection. Sample group 3 included stool samples from HIV positive patients attending integrated counselling and testing centre (ICTC) without any of the mentioned GI discomfort complaints. Sample group 4 included stool samples from apparently healthy asymptomatic subjects from the community without history of hospital admission or intake of any antibiotics/anti-parasitic drugs in last two weeks prior to the sample collection. Liver aspirate and stool sample from patients with well-defined liver abscesses greater than 5 cm diameter confirmed through abdominal ultrasound were considered in sample group 5 and sample group 6 respectively.

### Exclusion criteria

Specific and general exclusion criteria were considered as mentioned. For sample groups 1 and 2, stool from patients with chronic diarrhea, *Clostridium difficile* associated diarrhea, colitis or any other bacterial, fungal, viral or parasitic causes of diarrhea were excluded. Patients with associated co-morbidities like hepatitis, lactose intolerance or surgical emergencies like appendicitis were also excluded. In sample group 5, liver aspirate from patients with complications like impending abscess rupture, sepsis, hepatic malignancy were excluded. Besides, pregnant women, patients with malignancies, very sick patients and those unwilling to provide consent for the study in general were excluded.

### Sample collection

Stool samples were collected for groups 1, 2, 3, 4 and 6. Aspirates from liver abscesses were collected in group 5. Water samples from major sewage points in and around the hospital and various parts of the city were collected in sample group 7. Only single sample per case was included in the study of either nature.

### Sample processing

Stool samples were collected in wide-mouthed sterile containers without preservative and immediately transported to the microbiology laboratory for further processing. Aspirate from liver abscess was collected through ultrasonography guided fine needle aspiration in the gastroenterology department of the tertiary care hospital. Sewage water samples were collected in wide-mouthed bottles and filtered through a membrane filter of 1.2 μm pore size using a vacuum device. Further, washing, concentration and separation was performed as described previously. [15] All the samples were immediately processed for microscopy while a portion was stored at—80^0^ C for molecular studies.

### Microscopy

Direct microscopy was performed for all the stool samples, aspirates and sewage samples through wet, saline and iodine mount to screen for the presence of trophozoites/cysts of *Entamoeba* spp. In stool samples and sewage samples with no or low count of cysts, formol ether sedimentation was performed and re-examined by microscopy. Any sample was considered negative only if no cyst was found even after the concentration technique.

### Molecular detection of *Entamoeba* spp. from the samples

For the molecular detection of the *Entamoeba* spp. in the samples, DNA extraction was carried out using QIAGEN stool mini kit (Qiagen, Germany) as per the manufacturer’s instructions. The extracted DNA was stored at -20^0^ C till further processing.

Nested multiplex PCR was performed for the detection of *E*. *histolytica*, *E*. *dispar* and *E*. *moshkovskii* in all the samples using species specific primers targeting 16S like rRNA gene. [16] For screening of *E*. *bangladeshi*, conventional PCR with primers targeting 18S small subunit ribosomal RNA gene sequence was used. [17] Briefly 2.0 μL of 200 M concentrations of each of the deoxynucleoside triphosphates (dNTPs) (GeNei, Bangalore, India), 2.5 μL of 10X reaction buffer (GeNei, Bangalore, India), 0.3 μL of 5U Taq DNA Polymerase (GeNei, Bangalore, India) and 1 μL of each oligonucleotide primers were used to prepare reaction mixture. Five microliters (50 ng) of the DNA template and milli Q was added to maintain the final volume of 25 μL. The reaction mixture was subjected to PCR protocol as described previously. [16,17] Further, agarose gel electrophoresis was performed using 1.5% agarose gel with molecular marker of 100 bp ladder (GeNei, Bangalore, India) to examine the PCR products for their expected band size.

### Sequencing of PCR products

For species confirmation, ten randomly selected PCR products were subjected to DNA sequencing of the partial region of SSU rRNA gene. The forward and reverse primers were manually aligned. Further, with the help of Bioedit software a consensus sequence was created. The sequences were compared with the reference sequence from National Centre for Biotechnology Information using Basic Local Alignment Search Tool. The previously submitted sequences (Accession number: GenBank: X56991, GenBank: Z49256) were used as the reference sequences.

### Statistical analysis

Sample size in each group was calculated by using the formula provided by Kish with confidence interval at 95% and confidence level (α) = 0.05. [18]

n=(Zα2)p(1−p)d2

where n = required sample size, Z = value for level of confidence at α confidence level, p = expected prevalence, d = precision. In each group, more cases were recruited than required to increase the power of study and to eliminate any bias conclusion which may be driven otherwise in comparison with lower numbers. Data from questionnaire was entered in Microsoft Excel and cross-checked for accuracy. Association of the demographic factors with presence of *Entamoeba* was studied by univariate logistic regression analysis. In the analysis, the dependent variable was presence of *Entamoeba* spp. while the independent variables were the demographic and socioeconomic features. The samples from different groups were included for the analysis. The samples showing presence of any *Entamoeba* spp. either by microscopy or molecular detection were included as positive cases. Factors showing significant associations were further subjected to multivariate analysis. All statistical analysis was performed using 2019 Medcalc software (version: bvba, Ostend, Belgium). The level of statistical significance was set as p < 0.05.

## Results

### Microscopic examination

By microscopic examination in the stool samples, 26 (5.4%) cases showed the presence of cysts of *Entamoeba spp*. In the liver abscess aspirates, 8 (6.4%) samples showed presence of trophozoites of *Entamoeba* spp. through wet mount microscopy.

### Molecular detection

The prevalence of *Entamoeba* spp. in the different study groups through nested multiplex PCR method have been summarized in Table 1. In regard to the clinical manifestations, the overall prevalence of *Entamoeba* spp. in the intestinal cases, extra-intestinal cases and asymptomatic population was found to be 6.6% (20/301), 86.4% (108/125) and 10.5% (13/123) respectively. While *E*. *histolytica* was seen in the majority of the GI discomfort patients and in all the liver abscess patients, *E*. *dispar* was common in the asymptomatic population. All the samples with positive result on microscopy were also positive by molecular method. Sequencing of the ten amplicons confirmed the diagnosis of *E*. *histolytica* (7) and *E*. *dispar* (3) in the samples. Sewage water samples in group 7 did not show the presence of any *Entamoeba* spp. *E*. *moshkovskii* and *E*. *bangladeshi* was not found in any of the tested groups.

**Table 1:** Distribution of *Entamoeba* species in different study groups.

Study Population		Calculated sample size (n)	Collected sample size (n)	Positive by microscopy	Positive by PCR	Species	
						*E*. *histolytica* *n (%)*	*E*. *dispar* *n (%)*
Based on sample groups	Group 1	40	200	10 (5)	15 (7.5)	10 (5)	5 (2.5)
	Group 2	57	101	3 (2.9)	5 (4.9)	3 (2.9)	2 (1.9)
	Group 3	28	43	2 (4.6)	3 (6.9)	2 (4.6)	1 (2.3)
	Group 4	65	80	6 (7.5)	10 (12.5)	4 (5.0)	6 (7.5)
	Group 5	81	125	8 (6.4)	108 (86.4)	108 (86.4)	0
	Group 6	27	50	5 (10)	8 (16.0)	6 (12.0)	2 (4.0)
Based on clinical presentation*	Intestinal symptoms	-	301	13 (4.3)	20 (6.6)	13 (4.3)	7 (2.3)
	Liver abscess	-	125	8 (6.4)	108 (86.4)	108 (86.4)	0
	Asymptomatic	-	123	8 (6.5)	13 (10.5)	6 (4.8)	7 (5.6)

*Group 6 not included.

**Group 1**: patients with complaints of gastrointestinal (GI) discomfort **Group 2:** human immunodeficiency virus (HIV) positive patients attending the anti-retro viral therapy (ART) centre. **Group 3**: HIV positive patients attending integrated counselling and testing centre (ICTC) without any GI discomfort complaints. **Group 4**: asymptomatic subjects from the community **Group 5** and **Group 6**: patients with well-defined liver abscesses.

### Factors associated with presence of *Entamoeba* spp.

The general characteristics of the study participants, and the results of univariate analysis are shown in Table 2. Univariate analysis revealed uneducated subjects (p = 0.001), low economic conditions (p = 0.012), untreated drinking water (p = 0.005), consumption of raw vegetables (p = 0.025), not washing hands before meals (p = 0.0003) were significantly associated with presence of *Entamoeba* spp. The results of multivariate logistic regression analysis have been shown in Table 3. The multivariate analysis revealed that non-educated participants were at higher odds of *Entamoeba* spp. infection as compared to the educated participants (OR = 1.89; 95% CI = 1.26–2.82). It was also found that use of untreated drinking water and not washing hands before meals increased the participants’ odds for *Entamoeba* spp. infection by 1.75 and 1.92 times, respectively, compared with their counterparts.

**Table 2:** Univariate analysis of factors associated with presence of *Entamoeba* spp. (n = 549).

Variables		No. of cases examined	*Entamoeba* positive cases n (%)	Odds ratio	95% CI	p-value
Age	≥ 41 years	244	60 (24.5)	1.2	0.66–2.15	0.538
	18–40	216	62 (28.7)	1.48	0.82–2.66	0.187
	≤ 17 years	89	19 (21.3)	1		
Gender	Male	324	88 (27.1)	1.21	0.81–1.79	0.342
	Female	225	53 (23.5)	1		
Locality	Rural	352	84 (23.8)	0.76	0.51–1.14	0.192
	Urban	197	57 (28.9)	1		
Education	Uneducated	193	65 (33.6)	1.87	1.26–2.76	0.001*
	Educated	356	76 (21.3)	1		
Employment	No	189	43 (22.7)	0.78	0.52–1.18	0.255
	Yes	360	98 (27.2)	1		
Economic status	Low	201	64 (31.8)	1.64	1.11–2.42	0.012*
	Middle	348	77 (22.1)	1		
Drinking water	Untreated	279	86 (30.8)	1.74	1.17–2.57	0.005*
	Filtered/boiled	270	55 (20.3)	1		
Hand washing before meals	No	106	42 (39.6)	2.28	1.45–3.57	0.0003*
	Yes	443	99 (22.3)	1		
Consumption of raw vegetables	Yes	236	72 (30.5)	1.55	1.05–2.28	0.025*
	No	313	69 (22)	1		
Contact with domestic animals	Often	299	76 (25.4)	0.97	0.66–1.42	0.876
	Rare	250	65 (26)	1		

*p-value <0.05 (significant)

**Table 3:** Logistic regression model showing factors associated with the presence of *Entamoeba* spp.

Predictors	Coefficient (Standard error)	p-value	Odds Ratio	95% CI
Education (Uneducated)	0.638 (0.2)	0.001*	1.89	1.26–2.82
Economic status (Low)	0.274 (0.23)	0.237	1.31	0.83–2.07
Drinking water (Untreated)	0.564 (0.19)	0.004*	1.75	1.19–2.59
Consumption of raw vegetables (Yes)	0.354 (0.19)	0.07	1.42	0.97–2.09
Hand washing before meals (No)	0.655 (0.24)	0.006*	1.921	1.20–3.08

*p-value <0.05 (significant)

## Discussion

The present single centre study highlights the distribution of *Entamoeba* spp. in different clinical presentations of *Entamoeba* infections in this geographical region of India. While varying prevalence of *E*. *histolytica* and *E*. *dispar* was found among the different study groups, morphologically similar *E*. *moshkovskii* and *E*. *bangladeshi* was not found in any of the groups as confirmed by molecular methods. The identical morphology of these *Entamoeba* spp. often lead to over representation and unnecessary treatment with anti-amoebic drugs in clinical settings when diagnosis is based on microscopy alone. [19] Molecular method is more reliable mode of detection as compared to microscopy as it can differentiate between the morphologically similar species of *Entamoeba*. These findings are in agreement with other studies which recommend PCR to be a better detection tool for *Entamoeba* infections. [20,21] Based on clinical presentation, we found the prevalence of *Entamoeba* sp. more in asymptomatic population as compared to cases with intestinal symptoms. It is well recognized that 90% of the *Entamoeba* infections are asymptomatic. [4] However, contrary to our finding, few reports have shown prevalence of *Entamoeba* more in the stool of diarrheal and other GI discomfort patients as compared to the stool of healthy control subjects. [22,23] This difference can be attributed to the regional differences in the habits and living conditions of the participants. Moreover, *E*. *dispar* was the predominant species in the asymptomatic population in the present study.

Among GI discomfort patients, the prevalence of *E*. *histolytica* was more (5%) as compared to *E*. *dispar* (2.5%) in this study. A similar study conducted on GI discomfort patients in Iran showed comparable results. [24] On the contrary, a report from South India on the patients with comparable profile attending the tertiary care centre showed considerable prevalence of *E*. *dispar* (8.8%). [25] Other studies conducted in the northern part of the subcontinent on stool samples from gastrointestinal infections showed prevalence rate of *Entamoeba* spp. ranging from 7% to 12.2% through different detection methods such as microscopy, dot blot analysis and PCR. [25–27]

Numerous seroprevalence studies emphasize the fact that HIV positive individuals are at a risk of *Entamoeba* infections. [19,28] Studies have shown the presence of *Entamoeba* spp. in diarrheal as well as non-diarrheal asymptomatic HIV patients. [29–31] In Indian context, varying prevalence rate of 1.6% to 10.8% has been noted in HIV seropositive individuals in concordance with our findings. [19,32,33] A multicentric Indian study has shown higher prevalence of *E*. *histolytica* (6.17%) as compared to *E*. *dispar* (5.05%) in HIV positive individuals with/without diarrheal symptoms which is in-line with our results. [33]

*E*. *dispar* (7.5%) was found to be more prevalent than *E*. *histolytica* (5%) in the asymptomatic community population. A recent review on laboratory methods for detection of *Entamoeba* spp. has reported that 15–20% of the Indian population is infected with *E*. *histolytica*. [34] Another report from four states of India has shown higher prevalence of *E*. *histolytica* (13.7%) as compared to *E*. *dispar* (11.8%) and *E*. *moshkovskii* (7.8%) in the community. [35] Similarly, a study conducted on seven different settlements in Malaysia has shown the highest prevalence of *E*. *histolytica* (10.1%) followed by *E*. *dispar* (2.1%) and absence of *E*. *moshkovskii*. [36] The higher prevalence of *E*. *dispar* in the asymptomatic community population in our study could be attributed to the selection criteria of the study participants. Determination of the accurate prevalence of *Entamoeba* infections in community population is difficult as the mode of detection in endemic countries which is mostly microscopy is incapable of distinguishing between the morphologically similar species.

The present study showed a high prevalence of *E*. *histolytica* in the cases of liver abscess (86.4%). This was in agreement with a study from the adjoining area of Lucknow, India showing prevalence rate of 83.5%. [20] No other species of *Entamoeba* was found in the cases of liver abscess. In this context, in literature there is only one published report (abstract) from India which has shown the presence of both *E*. *dispar* and *E*. *moshkovskii* in the liver abscess aspirates concomitantly with *E*. *histolytica*. However, in the report, the authors have concluded the need for further validation of this finding. [37] As compared to liver aspirate samples (86.4%), *E*. *histolytica* was infrequently detected in the stool samples (16%) of the same patients. Previous studies have also reported that the screening of stool samples in liver abscess cases is not beneficial as often the number of parasites in stool is not detectable. [4]

In the lifecycle of this parasite, the mature cysts of *Entamoeba* are excreted through feces, and then they enter the sewage. *Entamoeba* can be present both in raw and treated wastewater samples. [38] However, in the present study none of the sewage water samples showed the presence of any *Entamoeba* spp. in concurrence with similar finding from other endemic countries. [39,40] The absence of *Entamoeba* spp. in sewage water samples could be attributed to the small volume of water collected for experimentation. Additionally, the presence of pharmaceuticals or their active metabolites that are excreted in feces and urine in the hospital sewage water can also affect the microbial load leading to decreased chances of detection. [41]

The main route of transmission of *Entamoeba* infection is feco-oral. The associated factors for *Entamoeba* infection specifically identified in the present study included use of untreated (not filtered/ not boiled) water and habit of not washing hands before meals as significant ones. Similar observations have been mentioned in many of the previous studies which report that poor handwashing practices in developing countries is a leading cause of infection with intestinal parasites. [42,43] Along with this, illiteracy, consumption of raw vegetables and poor economic conditions were also associated with *Entamoeba* infections similar to reports from other endemic countries. [44,45] The probable reasons for these associations could be the concomitant presence of poor hygiene and lack of sanitation along with these factors as exemplified in other related studies. [43,46]

The study was not without limitations. While it was a prevalence-based study limited to a single centre, only the molecular epidemiology of *Entamoeba* infections was studied. Nevertheless, the study carried out in one of the endemic locations in the Gangetic belt of India addresses several gaps in the existing epidemiological data on emerging *Entamoeba* spp. As epidemiology of parasites are ever evolving, this type of update is important to portray the current situation.

## Conclusions

The present study showed that *E*. *histolytica* remains the only species causing extra intestinal manifestations among the patients with liver abscess. Though the exact association of *E*. *dispar* in symptomatic intestinal presentations could not be affirmed, yet its presence raises a question on the pathogenicity of this organism. *E*. *moshkovskii* and *E*. *bangladeshi* were not detected in any of the cases. Molecular tools help in better detection and differentiation of *Entamoeba* spp.

## Supporting information

S1 Checklist STROBE Checklist. (DOCX)
