# Supplementary material for: Epidemiology of clinically relevant Entamoeba spp. (E. histolytica/dispar/moshkovskii/bangladeshi): A cross sectional study from North India
Source: PLoS Negl Trop Dis. 2021 Sep 7;15(9):e0009762. doi: 10.1371/journal.pntd.0009762 (PMC8448324; doi:10.1371/journal.pntd.0009762)
Supplement: S1 Checklist — (DOCX) [file pntd.0009762.s001.docx]

|  | **Item**  **No** | **Recommendation** | **Page**  **No.** | **Relevant text from manuscript** |
| --- | --- | --- | --- | --- |
| **Title and abstract** | 1 | (a) Indicate the study’s design with a commonly used term in the title or the abstract | 1 | Title |
|  |  | (b) Provide in the abstract an informative and balanced summary of what was done and what was found | 2 | Abstract |
| **Introduction** | | | | |
| **Background/rationale** | 2 | Explain the scientific background and rationale for the investigation being reported | 3, 4 | Introduction, Para 1 and 2 |
| **Objectives** | 3 | State specific objectives, including any prespecified hypotheses | 4 | Introduction, Para 2 |
| **Methods** | | | | |
| **Study design** | 4 | Present key elements of study design early in the paper | 4 | The present study was a hospital-based, cross-sectional study carried out from September 2016 to August 2019 |
| **Setting** | 5 | Describe the setting, locations, and relevant dates, including periods of recruitment, exposure, follow-up, and data collection | 4 | Material and Methods; Study site and groups |
| **Participants** | 6 | 1. Cohort study—Give the eligibility criteria, and the sources and methods of selection of participants. Describe methods of follow-up   Case-control study—Give the eligibility criteria, and the sources and methods of case ascertainment and control selection. Give the rationale for the choice of cases and controls  Cross-sectional study—Give the eligibility criteria, and the sources and methods of selection of participants | 5, 6 | Material and Methods; Inclusion and exclusion criteria. |
|  |  | 1. Cohort study—For matched studies, give matching criteria and number of exposed and unexposed   Case-control study—For matched studies, give matching criteria and the number of controls per cas | N/A | This was a cross-sectional study. |
| **Variables** | 7 | Clearly define all outcomes, exposures, predictors, potential confounders, and effect modifiers. Give diagnostic criteria, if applicable | 6 | Materials and methods; Study site and groups. |
| **Data sources/ measurement** | 8* | For each variable of interest, give sources of data and details of methods of assessment (measurement). Describe comparability of assessment methods if there is more than one group | 6 | Materials and methods; Sample processing |
| **Bias** | 9 | Describe any efforts to address potential sources of bias | 6 | In each group, more cases were recruited than required to increase the power of study and to eliminate any bias conclusion which may be driven otherwise in comparison with lower numbers. |
| **Study size** | 10 | Explain how the study size was arrived at | 5 | Sample size in each group was calculated by using the formula provided by Kish with confidence interval at 95% and confidence level (α) = 0.05 |
| **Quantitative variables** | 11 | Explain how quantitative variables were handled in the analyses. If applicable, describe which groupings were chosen and why | 8 | Statistical analysis. |
| **Statistical methods** | 12 | (a) Describe all statistical methods, including those used to control for confounding | 8 | Statistical analysis |
|  |  | (b) Describe any methods used to examine subgroups and interaction | N/A | There was no subgroup |
|  |  | (c) Explain how missing data were addressed | N/A | There was no missing data |
|  |  | 1. Cohort study—If applicable, explain how loss to follow-up was addressed   Case-control study—If applicable, explain how matching of cases and controls was addressed  Cross-sectional study—If applicable, describe analytical methods taking account of sampling strategy |  | The study subjects included patients attending different out-patient departments of Sir Sunderlal hospital, Varanasi. Additionally, volunteers from an adjoining area (Naria, Varanasi) in the immediate vicinity (within 1km) of the hospital were also included in the study. |
|  |  | (e) Describe any sensitivity analyses | N/A |  |
| **Results** | | | | |
| **Participants** | 13* | (a) Report numbers of individuals at each stage of study—eg numbers potentially eligible, examined for eligibility, confirmed eligible, included in the study, completing follow-up, and analyse | N/A | This was a cross-sectional study with only one stage |
|  |  | (b) Give reasons for non-participation at each stage | N/A |  |
|  |  | (c) Consider use of a flow diagram | N/A |  |
| **Descriptive data** | 14* | (a) Give characteristics of study participants (eg demographic, clinical, social) and information on exposures and potential confounder) |  | Table 2 shows all the demographic and socioeconomic factors included in the study. |
|  |  | (b) Indicate number of participants with missing data for each variable of interest | N/A | There was no missing data. |
|  |  | (c) Cohort study—Summarise follow-up time (eg, average and total amount) | N/A | This was a cross-sectional study. |
| **Outcome data** | 15* | Cohort study—Report numbers of outcome events or summary measures over time | N/A | This was a cross-sectional study. |
|  |  | Case-control study—Report numbers in each exposure category, or summary measures of exposure | N/A | This was a cross-sectional study. |
|  |  | Cross-sectional study—Report numbers of outcome events or summary measures |  | Table 2; Univariate analysis of factors associated with presence of *Entamoeba* spp. (n = 549)  Table 3; Logistic regression model showing factors associated with the presence of *Entamoeba* spp. |
| **Main results** | 16 | (a) Give unadjusted estimates and, if applicable, confounder-adjusted estimates and their precision (eg, 95% confidence interval). Make clear which confounders were adjusted for and why they were included |  | Table 2; Univariate analysis of factors associated with presence of *Entamoeba* spp. (n = 549)  Table 3; Logistic regression model showing factors associated with the presence of *Entamoeba* spp. |
|  |  | (b) Report category boundaries when continuous variables were categorized | N/A | There were no continuous variables. |
|  |  | (c) If relevant, consider translating estimates of relative risk into absolute risk for a meaningful time period | N/A | No estimation of relative risks in the study |
| **Other analyses** | 17 | Report other analyses done—eg analyses of subgroups and interactions, and sensitivity analyses | N/A | There were no subgroups |
| **Discussion** | | | | |
| **Key results** | 18 | Summarise key results with reference to study objectives | 9, 10 | Discussion, Para 1 |
| **Limitations** | 19 | Discuss limitations of the study, taking into account sources of potential bias or imprecision. Discuss both direction and magnitude of any potential bias | 12 | Discussion, Para 8 |
| **Interpretation** | 20 | Give a cautious overall interpretation of results considering objectives, limitations, multiplicity of analyses, results from similar studies, and other relevant evidence | 9 - 12 | Discussion |
| **Generalisability** | 21 | Discuss the generalisability (external validity) of the study results | 17 | Conclusions; The present study showed that *E. histolytica* remains the only species causing extraintestinal manifestations among the patients with liver abscess. Though the exact association of *E. dispar* in symptomatic intestinal presentations could not be affirmed, yet its presence raises a question on the pathogenicity of this organism. *E. moshkovskii* and *E. bangladeshi* were not detected in any of the cases. |
| **Other information** | | | | |
| **Funding** | 22 | Give the source of funding and the role of the funders for the present study and, if applicable, for the original study on which the present article is based | 13 | The author(s) received no specific funding for this work. |

*Give information separately for cases and controls in case-control studies and, if applicable, for exposed and unexposed groups in cohort and cross-sectional studies.
